# Transactions of the Wisconsin State Agricultural Society

**Published:** 1882-04

**Authors:** 


TRANSACTIONS OF THE WISCONSIN STATE AGRICULTURAL SOCIETY. Vol. XIX. Prepared by GEO. E. BRYANT, Secretary. Madison: 1881. 8vo. pp. 500.

This volume contains a number of papers and addresses on various subjects of interest to the farmer, among which may be mentioned the Diseases of Plants and Animals, as Connected with Bacteria, by Prof. J. C. Arthur; Ensilage, by I. C. Sloane; and numerous interesting and instructive articles.

